# Evaluation of pediatric rheumatology telehealth satisfaction during the COVID-19 pandemic

**DOI:** 10.1186/s12969-021-00649-4

**Published:** 2021-12-09

**Authors:** Lindsay N. Waqar-Cowles, John Chuo, Pamela F. Weiss, Sabrina Gmuca, Marianna LaNoue, Jon M. Burnham

**Affiliations:** 1 Division of Rheumatology, Children’s Hospital of Philadelphia, 3501 Civic Center Blvd., CTRB 1100.16, Philadelphia, PA 19104 USA; 2 Center for Pediatric Clinical Effectiveness, Children’s Hospital of Philadelphia Research Institute, Philadelphia, PA 19146 USA; 3 College of Population Health, Thomas Jefferson University, Philadelphia, PA 19107 USA; 4 Division of Neonatology, The Children’s Hospital of Philadelphia, Philadelphia, PA 19104 USA; 5 Department of Pediatrics, University of Pennsylvania Perelman School of Medicine, Philadelphia, PA 19104 USA; 6 PolicyLab, Children’s Hospital of Philadelphia Research Institute, Philadelphia, PA 19146 USA; 7 Center for Research and Scholarly Development, Vanderbilt University School of Nursing, Nashville, TN 37203 USA

**Keywords:** COVID-19, Patient satisfaction, Telehealth, Telehealth usability questionnaire, Pediatric rheumatology

## Abstract

**Background:**

During the Coronavirus disease 2019 pandemic, ambulatory pediatric rheumatology healthcare rapidly transformed to a mainly telehealth model. However, pediatric patient and caregiver satisfaction with broadly deployed telehealth programs remains largely unknown. This study aimed to evaluate patient/caregiver satisfaction with telehealth and identify the factors associated with satisfaction in a generalizable sample of pediatric rheumatology patients.

**Methods:**

Patients with an initial telehealth video visit with a rheumatology provider between April and June 2020 were eligible. All patients/caregivers were sent a post-visit survey to assess a modified version of the Telehealth Usability Questionnaire (TUQ) and demographic and clinical characteristics. TUQ total and sub-scale (usefulness, ease of use, effectiveness, satisfaction) scores were calculated and classified as “positive” based on responses of “agree” or “strongly agree” on a 5-point Likert scale. Results were analyzed using standard descriptive statistics and Wilcoxon signed rank testing. The association between demographic and clinical characteristics with TUQ scores was assessed using univariate linear regression.

**Results:**

597 patients/caregivers met inclusion criteria, and the survey response rate was 42% (*n* = 248). Juvenile idiopathic arthritis was the most common diagnosis (33.5%). The majority of patients were diagnosed greater than 6 months previously (72.6%) and were prescribed chronic medications (59.7%). The median total TUQ score was 4 (IQR: 4–5) with positive responses in 81% of items. Of the subscales, usefulness scores were lowest (median: 4, *p* < 0.001). Telehealth saves time traveling was the highest median item score (median = 5, IQR: 4–5). Within subscales, items that scored significantly lower included convenience, providing for needs, seeing rheumatologist as well as in person, and being an acceptable way to receive rheumatology services (all *p* < 0.001). There were no significant demographic or clinical features associated with TUQ scores.

**Conclusions:**

Our results suggest telehealth is a promising mode of healthcare delivery for pediatric rheumatic diseases but also identifies opportunities for improvement. Innovation and research are needed to design a telehealth system that delivers high quality and safe care that improves healthcare outcomes. Since telehealth is a rapidly emerging form of pediatric rheumatology care, improved engagement and training of patients, caregivers, and providers may help improve the patient experience in the future.

**Supplementary Information:**

The online version contains supplementary material available at 10.1186/s12969-021-00649-4.

## Background

In the wake of the Coronavirus disease 2019 (COVID-19) pandemic, ambulatory pediatric rheumatology healthcare delivery rapidly transformed to a mainly telehealth model at many centers using telephone and web-based video platforms [1]. Optimally used, telehealth may improve access to care, reduce time traveling to and from office visits, and improve cost-effectiveness. Potential drawbacks may include lower quality patient/clinician interactions and less accurate medical assessments [2, 3]. Given its widespread implementation and perceived feasibility [3], telehealth will be more commonly used in the future. However, the impact on patient satisfaction in pediatric rheumatology is unknown.

While telehealth has been examined in adult rheumatology and other pediatric subspecialties [4–6], studies characterizing and assessing pediatric rheumatology telehealth implementation are sparse and were conducted prior to the COVID-19 pandemic. In one study, families completed a survey that demonstrated a preference for in-person visits, but most respondents were not familiar with telemedicine as a modality [7]. A second study assessed differences in costs associated with telemedicine and found that pediatric rheumatology patients traveling to a remote site with a nurse-facilitator accrued lower costs and fewer hours of missed school and work [8]. Of eligible respondents receiving standard in-person care, only 42% expressed an interest in a telemedicine model [8]. To date, no studies have systematically evaluated patient/caregiver telehealth satisfaction in a diverse sample of pediatric rheumatology patients.

The purpose of this survey study was to evaluate pediatric rheumatology telehealth visits from the patient/caregiver perspective after telehealth implementation during the COVID-19 pandemic. Our specific objectives were to assess telehealth usefulness, ease of use, effectiveness, and satisfaction and identify clinical characteristics associated with overall satisfaction from the patient/caregiver perspective.

## Methods

### Study participants

All patients, both new and existing, with an initial telehealth video visit with a Children’s Hospital of Philadelphia (CHOP) rheumatology provider between 4/8/2020 and 6/30/2020 and had a valid email address in their medical record were eligible. Approximately 4% of patients did not have a valid email address, and therefore, did not receive the survey. Patients 18 years of age or older were allowed to complete the survey independently. Otherwise, a parent/caregiver completed the survey. This study was reviewed by the Children’s Hospital of Philadelphia Institutional Review Board (IRB) and was deemed not human subjects research since no protected health information was collected.

### Telehealth visit process

During the Spring of 2020, we developed a new telehealth visit process in response to the COVID-19 pandemic. Upon scheduling a video visit, a clinic coordinator ensured registration with the electronic health record (EHR) portal and distributed a standard set of pre-visit recommendations. Using the built-in telehealth capabilities of the health system’s EHR, the patient/family accessed the visit through the portal using a mobile phone, tablet, laptop, or desktop computer. The provider accessed the visit using either a mobile phone or tablet using mobile EHR functionality. Patient after-visit instructions were available through the EHR portal. Between 3/16/2020–4/13/2020, approximately 10% of visits were in-person, which included new patient and emergent patient visits. On 4/14/2020, new patient visits were also converted to telehealth, and thereby, received the survey.

### Survey administration

Within one day of completing the telehealth visit, all patients/caregivers were sent an anonymous post-visit Research Electronic Data Capture (REDCap©) telehealth satisfaction survey composed of a modified version of the Telehealth Usability Questionnaire (TUQ) and the Telemedicine Satisfaction and Usefulness Questionnaire (TSUQ) [9, 10]. Patient demographic and disease-related questions such as age, race, rheumatologic condition, diagnosis within past 6 months, and current rheumatologic medication(s) were included. After beginning survey administration, an additional question was added to differentiate between new and existing patients [see Additional file 1 for the complete survey]. The survey was automatically resent to patients/caregivers weekly up to three times until completed without the ability to skip questions.

### Data collection

The TUQ was chosen to assess telehealth usefulness, ease of use, effectiveness, reliability, and satisfaction. The TUQ has shown good internal consistency and independent content validity [9]. The TUQ includes 21 questions that are grouped into the five domains/sub-scales. Standardized Cronbach’s coefficient alpha values for the usefulness, ease of use, effectiveness, reliability, and satisfaction sub-scales are 0.85, 0.93, 0.87, 0.81, and 0.92, respectively [9]. The usefulness sub-scale gauges how the patient or parent perceives potential intended benefits of the telehealth system and whether the degree to which the healthcare interaction or service is similar to the in-person encounter. The ease of use sub-scale quantifies the patient or parent perception of how easy the telehealth system is to learn and whether the interaction with the telehealth interface is enjoyable. The effectiveness sub-scale assesses the quality of the telehealth interaction, specifically around audio and visual communication. Finally, the satisfaction sub-scale evaluates the patient’s overall satisfaction and willingness to use the system in the future [9]. We adapted the TUQ to include 14 questions focusing on our assessment priorities and eliminated telehealth platform questions less germane to our clinic-based improvement efforts. We substituted one item of the TUQ (“telehealth improves my access to health services”) with one item from the TSUQ (“video visits are a convenient form of healthcare delivery for me”) in the usefulness domain, since it assessed the information we were seeking to obtain from patients and families more clearly. Survey questions were delivered using a 5-point Likert scale with anchors strongly disagree [1] and strongly agree [5] and was only available in English.

### Survey analysis

Standard descriptive statistics were used to characterize the study population respondent demographic, disease- and medication related variables along with TUQ total and sub-scale scores. Likert scale responses of agree or strongly agree were classified as positive. Responses of neither agree or disagree, disagree, or strongly disagree were classified as neutral or negative. The Wilcoxon signed rank test was used to assess differences between individual survey items within each sub-scale and differences between total sub-scale scores. Univariate linear regression models were used to assess the association of demographic and underlying diagnosis with TUQ total and sub-scale scores. Bonferroni correction was used in the exploratory analysis of differences across clinical characteristics given there were multiple comparisons. Analyses were performed using IBM SPSS Statistics 20.0.

## Results

### Patient characteristics

We received responses from 42% (248/597) of patients and caregivers. The demographic, disease- and treatment characteristics, and visit type are detailed in Table 1. The most common rheumatic condition represented was juvenile idiopathic arthritis (JIA, 33.5%). Most were diagnosed with a rheumatologic condition greater than six months prior to the survey (76.2%) and were taking a medication prescribed by the rheumatologist (59.7%). Of those on medication, steroids were prescribed for 14.5%. Characteristics of the non-responders were not assessed, since the survey was anonymous.

**Table 1:** Patient demographics, disease, and treatment characteristics

Variable	***N*** = 248^**a**^
**Patient Age**	
≤ 5 years	35 (14.1%)
6–12 years	68 (27.4%)
13–17 years	118 (47.6%)
≥ 18 years	27 (10.9%)
**Race**	
Caucasian	188 (75.8%)
African American	16 (6.5%)
Asian	17 (6.9%)
Other	27 (10.9%)
**Condition**	
Juvenile idiopathic arthritis	83 (33.5%)
Systemic lupus erythematosus	16 (6.5%)
Inflammatory disease^b^	40 (16.1%)
Amplified musculoskeletal pain	36 (14.5%)
Other/Unknown	73 (29.4%)
Rheumatologic diagnosis in the past 6 months	59 (23.8%)
Prescribed rheumatologic medication(s)	148 (59.7%)
Glucocorticoid therapy for rheumatologic condition	36 (14.5%)
Rheumatology new patient visit (*n* = 212)	85 (40.1%)

^a^Unless otherwise indicated

^b^Diagnoses included in the “inflammatory disease” category include Sjogren syndrome, vasculitis, juvenile dermatomyositis, chronic nonbacterial osteomyelitis, idiopathic uveitis, localized scleroderma, systemic sclerosis, Behcet syndrome, sarcoidosis, and periodic fever syndrome or auto-inflammatory disorder

### Assessment of Total TUQ and sub-scale scores

**Fig. 1:**
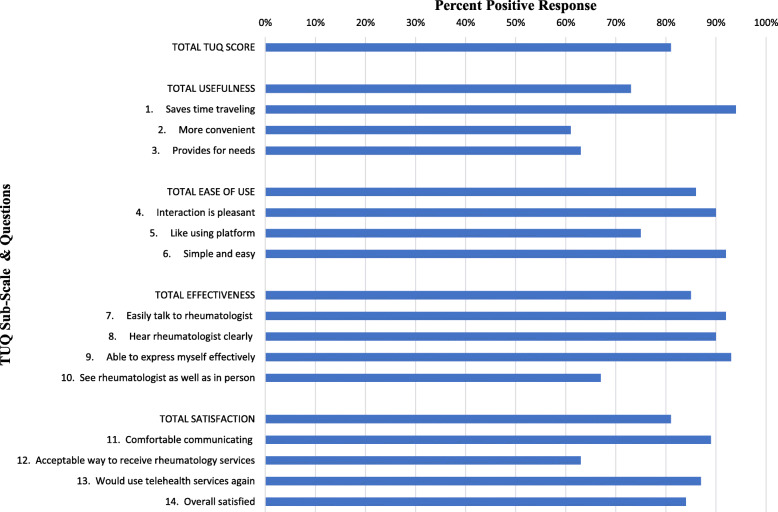
Graphic Depiction of Positive Response by Telehealth Usability Questionnaire (TUQ)

We graphically depicted positive responses across the total TUQ and subscales (Fig. 1). There was no statistically significant difference between positive responses of agree and strongly agree or negative responses of disagree or strongly disagree.

We then assessed the total TUQ and subscale scores to identify the determinants of the telehealth satisfaction (Table 2). The median total TUQ score was 4 (IQR: 4–5). Patients and caregivers provided positive responses in 81% of items. Of the subscales, usefulness scores were lowest (*p* < 0.001).

**Table 2:** Total TUQ and Sub-Scale Scores

	Positive Response^**a**^ N (%)	Neutral / Negative Response N (%)	Mean (SD)	Median (IQR)
**Total TUQ Score**	201 (81%)	47 (19%)	4.18 (0.65)	4 (4–5)
**Usefulness**	181 (73%)	67 (27%)	4.04 (0.72)	4 (4–5) ^b^
1. Saves time traveling	233 (94%)	15 (6%)	4.62 (0.61)	5 (4–5)
2. More convenient	151 (61%)	97 (39%)	3.77 (1.06)	4 (3–5) ^c^
3. Provides for needs	156 (63%)	92 (37%)	3.75 (0.95)	4 (3–4) ^c^
**Ease of Use**	213 (86%)	35 (14%)	4.29 (0.69)	4 (4–5)
4. Interaction is pleasant	223 (90%)	25 (10%)	4.42 (0.74)	5 (4–5)
5. Like using platform	186 (75%)	62 (25%)	4.02 (0.95)	4 (3–5) ^d^
6. Simple and easy	228 (92%)	20 (8%)	4.40 (0.70)	5 (4–5)
**Effectiveness**	211 (85%)	37 (15%)	4.26 (0.77)	5 (4–5)
7. Easily talk to rheumatologist	228 (92%)	20 (8%)	4.42 (0.84)	5 (4–5)
8. Hear rheumatologist clearly	223 (90%)	25 (10%)	4.37 (0.92)	5 (4–5)
9. Able to express myself effectively	231 (93%)	17 (7%)	4.50 (0.69)	5 (4–5)
10. See rheumatologist as well as in person	166 (67%)	82 (33%)	3.77 (1.14)	4 (3–5) ^e^
**Satisfaction**	201 (81%)	47 (19%)	4.12 (0.74)	4 (4–5)
11. Comfortable communicating	221 (89%)	27 (11%)	4.31 (0.75)	4 (4–5)
12. Acceptable way to receive rheumatology services	156 (63%)	92 (37%)	3.72 (1.09)	4 (3–5) ^f^
13. Would use telehealth services again	216 (87%)	32 (13%)	4.26 (0.81)	4 (4–5)
14. Overall satisfied	208 (84%)	40 (16%)	4.20 (0.78)	4 (4–5)

^a^ Positive response includes “agree” or “strongly agree”, while neutral/negative response includes “Neither agree nor disagree”, “disagree” and “strongly disagree”

^b^ Median statistically significantly lower compared with ease of use, effectiveness, and satisfaction sub-scales

^c^ Medians statistically significantly lower compared with item 1

^d^ Median statistically significantly lower compared with items 4 and 6

^e^ Median statistically significantly lower compared with items 7, 8, and 9

^f^ Median statistically significantly lower compared with items 11, 13, and 14

We next assessed differences in domain-specific responses. Within the usefulness domain, a high proportion attested to telehealth visits saving time (median = 5, IQR: 4–5). However, scores for telehealth visits being more convenient (median = 4, IQR: 3–5) and providing for rheumatology needs (median = 4, IQR: 3–4) were significantly lower (*p* < 0.001 for both). Within the ease of use domain, a high proportion found the interaction pleasant and simple and easy (median = 5, IQR: 4–5 for both). Significantly lower scores were noted for “like using platform” (median = 4, IQR: 3–5); *p* < 0.001). Within the effectiveness domain, verbal communication items all received high scores (median = 5, IQR: 4–5), but the visual communication scores (median = 4, IQR: 3–5) were significantly lower (*p* < 0.001). Within the satisfaction domain, patients and caregiver scores for comfort communicating, future use, and overall satisfaction were high (median = 4, IQR: 4–5). However, when asked whether telehealth is an acceptable way to receive rheumatology services, scores (median = 4, IQR: 3–5) were significantly lower (*p* < 0.001).

### Clinical characteristics and satisfaction

**Fig. 2:**
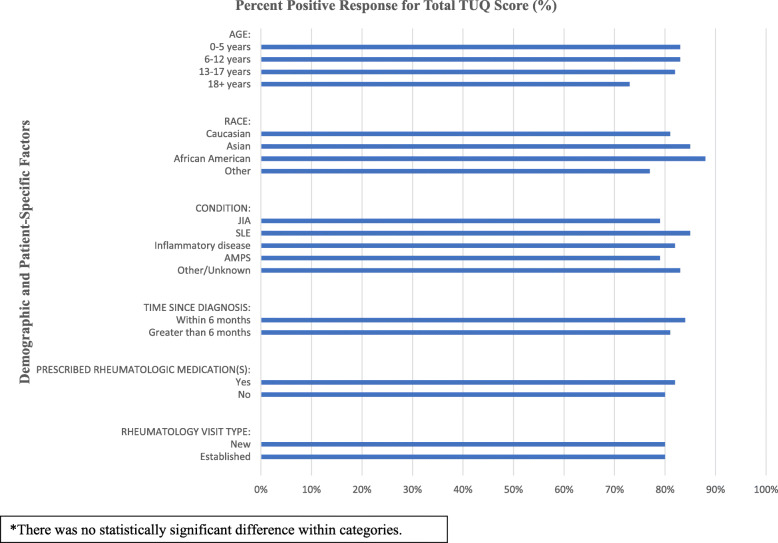
Positive Telehealth Satisfaction Across Demographic and Patient-Specific Factors

Descriptive analysis of positive responses by demographic and clinical features across total and sub-scale TUQ scores are depicted in Table 3. We graphically depicted positive responses for total TUQ across demographic and patient-specific factors (Fig. 2).

**Table 3:** Positive telehealth satisfaction according to demographic and patient-specific factors

Variable	Total Score	Usefulness	Ease of Use	Effectiveness	Satisfaction/ Future Use
**Age**					
0–5 years	83%	65%	90%	90%	85%
6–12 years	83%	73%	87%	87%	86%
13–17 years	82%	76%	86%	85%	80%
18+ years	73%	69%	75%	76%	70%
**Race**					
Caucasian	81%	73%	86%	84%	81%
African American	88%	77%	90%	97%	88%
Asian	85%	79%	86%	90%	84%
Other	77%	62%	84%	86%	76%
**Condition**^**a**^					
JIA	79%	68%	85%	84%	80%
SLE	85%	81%	88%	88%	83%
Inflammatory disease^b^	82%	73%	89%	84%	82%
AMPS	79%	76%	78%	83%	78%
Other/Unknown	83%	74%	88%	89%	82%
**Time since diagnosis**					
Within 6 months	84%	76%	89%	86%	84%
Greater than 6 months	81%	72%	85%	85%	80%
**Prescribed rheumatologic medication(s)**					
Yes (*n* = 148)	82%	73%	87%	87%	82%
Glucocorticoid therapy	82%	75%	88%	84%	81%
No glucocorticoid therapy	83%	73%	87%	87%	83%
No	80%	72%	84%	84%	79%
**Rheumatology visit type (*n*** **= 212)**					
New	80%	70%	86%	86%	79%
Established	80%	72%	83%	84%	80%

Positive satisfaction defined as responses of “agree” and “strongly agree”

^a^JIA: juvenile idiopathic arthritis; SLE: systemic lupus erythematosus; AMPS: amplified musculoskeletal pain syndrome

^b^Diagnoses included in the “inflammatory disease” category include Sjogren syndrome, vasculitis, juvenile dermatomyositis, chronic nonbacterial osteomyelitis, idiopathic uveitis, localized scleroderma, systemic sclerosis, Behcet syndrome, sarcoidosis, and periodic fever syndrome or auto-inflammatory disorder

The univariate regression model did not reveal differences in the telehealth satisfaction total or subscale scores according to age, race, rheumatologic condition, disease duration, glucocorticoid therapy or visit type.

## Discussion

We completed a survey assessing telehealth satisfaction in a diverse sample of pediatric rheumatology patients during the COVID-19 pandemic. This is the first study of its type and is timely given the rapid and widespread telehealth implementation in the United States. The study is strengthened by the use of validated telehealth usability instruments. While overall satisfaction with video visits was high, our data suggest that purposeful efforts will be required to design a care experience to meet the needs of patients and families.

There are encouraging signs that telehealth may be a viable method to deliver pediatric rheumatology care in the future. Across all telehealth assessment sub-scales, the majority of patients and caregivers reported positive experiences. A high proportion considered video visits to be time saving as well as easy to learn and use. We did not identify significant differences in the satisfaction based on rheumatologic diagnoses (e.g. JIA or amplified musculoskeletal pain syndrome), medication treatments, or demographic factors, which should be promising for communities where access to pediatric rheumatology providers is limited.

Although generally well-received, there was a lower percentage of patients and caregivers who endorsed the visits as more convenient or as an “acceptable” way to receive pediatric rheumatology care. These findings are consistent with a previous survey performed at a pediatric rheumatology center demonstrating that in-person visits were preferred over telehealth regardless of the need for travel. However, patients and families who had more familiarity with telehealth care were more likely to consider it equal or preferable to in-person visits [7]. Acceptability may increase with greater exposure to video visits and application in more targeted use cases. In addition, our study was performed during a rapid implementation of video visits at the beginning of the COVID-19 pandemic. It is possible that acceptability will increase when pediatric rheumatology providers have more familiarity with telehealth delivery and identify more optimal use cases. Research and innovation are needed to determine how evolving telehealth technology can help providers perform virtual exams with high fidelity and safety, thereby improving pediatric rheumatology telehealth delivery [11].

Previous studies of patient satisfaction may help identify methods to improve telehealth implementation. While little has been published on the specific drivers of patient satisfaction with in-person pediatric rheumatology care, drivers of a positive patient experience in the pediatric literature include the efficiency of the visit and more important, communication between team members and between the patient, caregivers, and healthcare provider [12–14]. Within the realm of telehealth, the drivers of patient satisfaction are less defined. A systematic review of patient satisfaction with telehealth demonstrated its potential, documenting that it was convenient, easy to use, enhanced communication, and in some cases, improved outcomes [15]. In the included research studies, however, telehealth was mainly used to augment management in specific chronic diseases (e.g. sleep apnea) or support specific clinical functions (e.g. insulin therapy monitoring). Broader applications to general populations such as ours are less well studied. Pediatric subspecialty-specific guidance to enhance telehealth satisfaction has been published for gastroenterology and rheumatology [16, 17]. The authors suggest that close attention to the pre-visit process, patient-facing technology logistics, visit location, history and physical exam completeness, and post-visit recommendations are critical to achieve a positive patient experience. Provider and staff training will undoubtedly be required to perform these functions optimally.

Improved clinical evaluation tools and enhanced understanding of telehealth capabilities are needed to arrive at the ideal telehealth encounter for all pediatric rheumatology patients. Patient reported outcomes (PROs) are being increasingly used as clinical measures to assess disease activity in pediatric rheumatology. The Juvenile Arthritis Disease Activity Score (JADAS), for example, combines patient reported values with physician and physical exam values to calculate disease state in JIA [18]. Telehealth visits pose a challenge to the standard physical exam, which has been superior in care delivery. Therefore, alternative approaches may be needed to augment the clinical exam to improve satisfaction with the telehealth visit. Examples may include use of the Juvenile Arthritis Multidimensional Assessment Report (JAMAR) for patient-reported assessment of disease, the video paediatric gait arms legs and spine (v-pGALS) for virtual musculoskeletal examination, and personal devices such as blood pressure monitors and symptom trackers [17, 19]. Additionally, it will be important to address equity issues related to internet and hardware access, insurance status, limited language proficiency, and health literacy in future quantitative and qualitative work to ensure the telehealth experience is optimal for all patients requiring pediatric rheumatology care [20].

Our study had several limitations to consider. Given the telehealth platform was rapidly deployed and changed during the study period, improved training and implementation for patients, caregivers, and providers may help increase satisfaction in the future. Additionally, responses may have been influenced by the social context of the COVID-19 pandemic and therefore may not be generalizable. We received responses from 42% of patients and caregivers who may represent a more engaged cohort and be less representative of the pediatric rheumatology population. Since we did not have access to personal health information, we were unable to compare the demographics of patients that responded to the survey to those that did not respond. In addition, those unable to access care during the pandemic and those without an active email address were not included in the study. Therefore, it is possible that we are capturing a group of patients that were more likely to be satisfied with telehealth. For example, patients may have canceled or not participated in telehealth visits and would not have received a satisfaction survey. Also, the survey was only available in English, which may have excluded non-English speakers from responding. There was a brief period during survey administration where the type of visit was not assessed. However, analysis of visit type and satisfaction showed no significant difference indicating that it was appropriate to include all of the data together. There may be additional constructs of satisfaction with telehealth, such as technological reliability, that were not addressed by our survey that could benefit from future analysis. Finally, the physician perspective regarding usability of and satisfaction with telehealth for specific visit-level processes and patient populations was not assessed and would be impactful to include in future research studies.

## Conclusions

These data provide the foundation for understanding patient and caregivers’ satisfaction with telehealth in pediatric rheumatology and suggest that it is a promising mode of healthcare delivery. Further work is needed to design specific telehealth processes for pediatric rheumatology populations and assess outcomes. Given that telehealth is a rapidly emerging form of pediatric rheumatology care, we will need to engage patients, caregivers, multidisciplinary healthcare providers, and our health network technology partners to design interventions to streamline care to improve patient satisfaction and overall experience.

## Supplementary Information

**Additional file 1.** Telehealth Satisfaction Survey. This is a copy of the survey which was completed by patients and caregivers.

## Data Availability

The datasets used and/or analyzed during the current study are available from the corresponding author upon reasonable request.

## Abbreviations

WHO: World Health Organization
CHOP: Children’s Hospital of Philadelphia
IRB: Institutional Review Board
REDCap: Research Electronic Data Capture
TUQ: Telehealth Usability Questionnaire
JIA: Juvenile Idiopathic Arthritis
AMPS: Amplified Musculoskeletal Pain Syndrome
IBD: Inflammatory Bowel Disease
JADAS: Juvenile Arthritis Disease Activity Score
JAMAR: Juvenile Arthritis Multidimensional Assessment Report
v-pGALS: Video Paediatric Gait Arms Legs and Spine
