# Supplementary material for: Evaluation of pediatric rheumatology telehealth satisfaction during the COVID-19 pandemic
Source: Pediatr Rheumatol Online J. 2021 Dec 9;19:170. doi: 10.1186/s12969-021-00649-4 (PMC8655491; doi:10.1186/s12969-021-00649-4)
Supplement: Supplementary file 1 — Additional file 1. Telehealth Satisfaction Survey. This is a copy of the survey which was completed by patients and caregivers. [file 12969_2021_649_MOESM1_ESM.docx]

| **Additional File 1. Telehealth Satisfaction Survey.** | |
| --- | --- |
| **Total Usefulness*** | |
| 1. Telehealth saves me time traveling to my child’s rheumatology visit. | |
| 2. Telehealth is a more convenient form of rheumatology care for my child. | |
| 3. Telehealth provides for my child’s rheumatology needs. | |
| **Total Ease of Use** | |
| 4. The way I interact with telehealth is pleasant. | |
| 5. I like using the telehealth system. | |
| 6. The telehealth system is simple and easy to understand. | |
| **Total Effectiveness** | |
| 7. I could easily talk to my child’s rheumatologist using the telehealth system. | |
| 8. I could hear my child’s rheumatologist clearly using the telehealth system. | |
| 9. I felt I was able to express myself effectively. | |
| 10. Using the telehealth system, I could see my child’s rheumatologist as well as if we met in person. | |
| **Total Satisfaction** | |
| 11. I feel comfortable communicating with my child’s rheumatologist using the telehealth system. | |
| 12. Telehealth is an acceptable way to receive rheumatology services. | |
| 13. I would use telehealth services again. | |
| 14. Overall, I am satisfied with this telehealth system. | |
| 15. Was today’s telehealth visit your first appointment with CHOP Rheumatology? | Yes  No |
| 16. What is your/your child’s current age? | 0-2 years  3-5 years  6-12 years  13-17 years  18 years or older |
| 17. What is your/your child’s race? | American Indian  Asian  Caucasian  Native Hawaiian  Black or African America  Other |
| 18. Please check the name of the condition you/your child is seeing the rheumatologist for today. | Juvenile Idiopathic Arthritis (JIA)  Enthesitis  Lupus or Mixed Connective Tissue Disorder  Sjogren Syndrome  Vasculitis  Juvenile Dermatomyositis (JDM)  Chronic Nonbacterial Osteomyelitis (CNO)/Chronic Recurrent Multifocal Osteomyelitis (CRMO)  Idiopathic Uveitis  Localized Scleroderma  Systemic Sclerosis  Behcet Syndrome  Periodic fever syndrome or Auto-inflammatory Disorder  Sarcoidosis  Mechanical pain (e.g. patellofemoral syndrome)  Amplified Musculoskeletal Pain Syndrome (AMPS  Other  I Don’t Know |
| 19 Does the rheumatologist prescribe any mediation for you/your child’s condition? | Yes  No |
| 20. If the rheumatologist prescribes medication, does it include steroids? | Yes  No |
| 21. Were you/your child diagnosed with the primary rheumatic condition within the past 6 months? | Yes  No |
| *Items 1-14 were scored on a 5-point Likert scale where strongly disagree=1, disagree=2, neither agree nor disagree=3, agree=4, strongly agree=5.  Sub-Scale Definitions:  Usefulness: how the system functions to provide a healthcare interaction/service similar to the in-person encounter (3 items)  Ease of use: how easy the system is to learn and use to facilitate rapid work completion (3 items)  Effectiveness: how pleasant the system is to use and the quality of the interaction compared to the in-person encounter (4 items)  Satisfaction: overall satisfaction and willingness to use the system in the future (4 items) | |
